# Nexus between stock markets, economic strength, R&D and environmental deterioration: new evidence from EU-27 using PNARDL approach

**DOI:** 10.1007/s11356-022-24458-8

**Published:** 2022-12-06

**Authors:** Muhammad Mushafiq, Błażej Prusak

**Affiliations:** Faculty of Management and Economics, Gdansk University of Technology, Gdansk, Poland

**Keywords:** Stock market indices, Economic strength, Research and development, Environmental deterioration, Sustainable development, Asymmetry

## Abstract

This research investigates the impact of stock market indices, economic strength, and research and development expenditures on environmental deterioration in the EU-27 countries for the period 2000–2020. This study utilized linear and non-linear panel ARDL to estimate the short- and long-run effect. According to the results, the stock market indices have negative effect on environmental deterioration in the symmetric form. However, the asymmetric evidence shows that in the long run the positive shocks of stock market indices contribute positively to the environmental deterioration and negative shocks decrease the environmental deterioration. This effect is reversed in the short run. Linear effect of economic strength on environmental deterioration is positive. For non-linear effect, the long-run shocks show no difference. However, the negative shock of economic strength in the short run causes an increase in the environmental deterioration. Symmetric evidence for research and development increases environmental deterioration. However, asymmetric results show weak evidence. The study has policy implications in context of achieving sustainable development goals.

## Introduction

Climate change and environmental deterioration have been called some of the biggest challenges humanity has faced in recent years. The United Nations, through its 2030 Agenda for Sustainable Development, reaffirms its resolve to safeguard the world from degradation by promoting environmentally responsible consumption and production, ensuring the responsible use of the planet’s limited natural resources, and combating climate change as quickly as possible to meet the needs of both current and future generations. Both the production and burning of fossil fuels result in carbon emissions and air pollution, contributing to global warming. Researchers, economists, and politicians are devoting more time and effort than ever before to finding ways to reduce carbon dioxide emissions while simultaneously fostering economic growth and the expansion of financial markets.

This is in response to the fact that climate change is having a negative impact on the course of economic strengthening of financial markets. For instance, a growing body of research examines the correlation between country stock market performance, GDP, and environmental quality. These studies show that the growth of the financial markets may have contrasting effects on environmental sustainability in various locations and nations (Mhadhbi et al. 2021; Paramati et al. 2017; Zafar et al. 2019; Zhao and Yang 2020).

Scientists agree that cutting carbon emissions is essential if we are going to have any chance of halting global warming. A significant portion of the scientific investigation into climate change is underpinned by scientific and technical principles. An economic and financial market–based method that focuses on evaluating the causation between returns on the stock market and carbon emissions caused by fossil fuels might also make valuable contributions to the effort to solve the problem of carbon emissions (Chang et al. 2020).

This approach would investigate the relationship between the two variables. An inverse relationship between stock market development and carbon dioxide emissions is essential for this strategy to work. Financial choices that result in better stock returns and fewer carbon emissions would be encouraged as a result of this unfavorable impact. Stock market developments provide investors accessibility to more funding options, such as equity financing, which might result in increased investment in sustainable energy projects (Minier 2009; Paramati et al. 2016; Sadorsky 2010, 2012). Since this is the case, it follows that changes in the stock market growth plays significant role in cutting down on carbon emissions.

A central tenet of the endogenous growth school of thought is that increased spending on R&D can boost production and resource utilization efficiency. The ability to invest in research and development and, by extension, to adopt effective technologies improves as national incomes rise. Technologies that are more resourceful and produce less pollutants and waste products are better for the environment as a whole (Dinda 2004; Komen et al. 1997). Spending more on R&D, for example, can improve ecological integrity if effective environmental management systems are in place to control waste output.

Due to the scale effects of increased production that accompany higher growth and trade openness, research and development (R&D) might have major influence on environmental condition (Castellani and Pieri 2013; Freimane and Bāliņa 2016; Minniti and Venturini 2017). Even though modern technology has the potential to raise efficiency, increasing productivity may still require the use of additional natural resources, which may result in greater carbon emissions (Awaworyi Churchill et al. 2019). The fact that there are potentially dwindling benefits to investing in R&D over time only adds weight to this idea.

Companies rely heavily on capitalization in the stock market because it allows them to borrow money at low interest rates and invest in environmentally friendly technology. It creates openings for businesses to adopt more sustainable practices and renewable energy sources. Increased economic activity using older, less energy-efficient technologies is the primary cause of an initial rise in emissions when stock market value rises. However, when trust among investors grows, businesses may access cheaper funding, which has a beneficial effect on the environment through the widespread use of green technologies. There will be less spending and fewer emissions as a result of the confidence of investors and the availability of cheap financing. Given that a company’s limited resources prevent it from investing in environmentally friendly technologies, the stock market’s buying power has the potential to make a major impact in addressing the problem of carbon emissions by employing environmentally friendly means (Azeem et al. 2022).

There are a variety of different conclusions that may be drawn from the research done on the link between industrialization and the degradation of the natural environment. According to the findings of Paramati et al. (2018), developed economies’ stock markets are associated with lower emissions. On the other hand, Zakaria and Bibi (2019) find that the growth of the stock market drives up emissions. In principle, the effect that stock market development has on carbon emissions can vary quite a bit, and this is partly due to the fact that the effectiveness of the financial environment plays a significant role in the matter. However, there are not any clear answers on which clear conclusions can be drawn; therefore, this study is motived to explore the effect of stock market growth, economic strength, and R&D expenditures’ impact on the environmental degradation in the EU-27.

The motivation of this study is driven by the work of Shahbaz et al. (2020a, b) as they explored the symmetric nexus in the context of the UK. This study’s contribution to the literature and objectives are overlapping: (1) the empirical exploration of stock market indices, economic strength, and research and development’s impact on the environmental deterioration, (2) measuring above-mentioned variables on the environmental deterioration, (3) extending the previous literature (Azeem et al. 2022; Chang et al. 2020; Mhadhbi et al. 2021; Paramati et al. 2018) to a full panel of EU-27 countries, (4) applying panel non-linear autoregressive distributed lag (PNARDL) to gauge the asymmetric relationship between the variables, and (5) providing spatial and temporal graphic evidence for better understanding of the relationship.

## Literature review

### Financial markets and environment

According to the finance-led growth theory, increased financial resources drive economic expansion, which in turn requires more energy and worsens environmental conditions (Pineiro-Chousa et al. 2017). Investors often interpret an increase in stock price as a sign of economic growth. When the stock market grows, company owners have easier access to capital, which allows them to broaden their activities (Sadorsky 2010). Investors and companies may be better able to spread their risks with the rising stock market activity (Mushafiq 2021). Because of this, it is expected that the carbon emissions would increase with the higher level of production.

The increase in economic activity and demand is a direct effect of the stock market’s rise. According to Dauda et al. (2021), traditional or outdated technology is to blame for environmental deterioration, and cutting-edge solutions are required to tackle today’s pressing environmental problems. These claims lay the groundwork for studying how the size of the stock market correlates with carbon dioxide emissions (Ozturk and Acaravci 2013; Sadorsky 2010; Zhang 2011). These points of view clarify the theoretical connection between business actions, energy requirements, and stock market growth. However, the correlation between stock market growth and carbon emissions has been the subject of few research. The expansion of the market has been linked to reduced carbon emissions per person, as discovered by Tamazian et al. (2009).

Capitalizing on the stock market is an efficient way to pool resources and cut down on financing expenses since it allows for optimized capital structure, financing major projects, simple direct and indirect financing, risk sharing, and access to cutting-edge technology. The most important gain from the stock market is the funding of research and development into cutting-edge, low-carbon emission technologies that help to slow the rate of environmental damage. As a result, stock market growth provides both developed and developing countries with cutting-edge and eco-friendly technologies that can boost energy efficiency and aid in the transition to more sustainable and environmentally friendly production methods, thereby lowering their carbon footprints (Claessens and Feijen 2006; Tamazian et al. 2009).

Some of the work is focused on the relationship of stock markets and environmental deterioration through energy usage. Sadorsky (2010) analyzes the connection between stock market growth and energy use by using panel data from 22 developing nations. The results suggest that the growth of the financial markets has a direct impact on the demand for energy in developing countries. Sadorsky (2011) utilizes the panel generalized method of moments (GMM) regression methodology to investigate the effect of stock market growth on energy consumption in nine emerging markets in Eastern and Central Europe. The study found that stock market turnover was the only factor that significantly and positively affected energy use. Çoban and Topcu (2013) found that among the original EU member states, growth of financial development were associated with much higher rates of household energy use. Whether one examines banking or stock market developments, the impact remains the same.

Others explored this aspect through the carbon emission. For instance, Zhang (2011) utilizes the same stock market data as Sadorsky’s (2010) study but applies time series analytic methods to examine the connection between China’s financial growth and carbon emissions. The results reveal that the efficiency of the Chinese stock market has less impact on carbon emissions. On the other hand, research by Tamazian et al. (2009) link improved access to finance with lower levels of carbon emissions per person. This research adds to the growing body of evidence that robust capital markets are crucial to both short- and long-term growth of companies by mitigating their exposure to liquidity risk and fueling technological innovation.

In addition, Paramati et al. (2018) investigated the impact of the financial markets on environmental degradation in both established and developing economies, taking into account energy efficiency, economic development, and population density. They tracked stock market growth through various criteria. Their research showed that leading stock market indicators were significantly negatively connected with carbon emissions in industrialized economies, whereas leading financial market indicators were favorably correlated with carbon emissions in the case of developing markets.

Recent research by Zeqiraj et al. (2020) examined the importance of technical innovation and renewable energy in low-carbon economies across EU member nations from 1980 to 2016, as well as their dynamic relationship with stock market growth and carbon emissions. In their analysis, they looked at changes in market capitalization to see how the stock market was doing over time. They proved that, over the long term, growth in the stock market leads to a higher intensity of carbon emissions. However, their work was limited to the 23 states of EU. Results from the study of long-run elasticities, in particular, indicate that both positive and negative shocks to stock market indicators might lower environmental integrity by raising carbon emissions Mhadhbi et al. (2021). Based on the evidence from the studies, it is evident that the relationship between stock markets and environmental deterioration is not conclusive. Therefore, this study explores the relationship between stock markets and environmental deterioration.

### Economic strength and environment

According to the sustainable development theory by Daly (1990), it presented the concept for sustainable development as a practical approach, explaining it theoretically by modeling J. R. Hicks’ concept of income and arguing that the notion of income as the maximum amount that an individual or a country could spend for a given time span and still be in the same financial position only at end of the term has sustainability incorporated into it. Furthermore, a reason was given that the practical rationale for measuring income is to have a guideline for how much one might consume year by year without being impoverished. Maximum sustainable consumption equals income.

There is no question that economies need to make economic growth in order to reduce the level of poverty and improve their infrastructure over the long term. However, a faster growth rate that is the outcome of more economic activity calls for a larger use of energy. When compared to the usage of renewable energy sources, a greater reliance on non-renewable energy sources, such as coal, crude oil, and natural gas, in commercial development may lead to the damage of the surrounding environment as a consequence of an increase in carbon emissions. To put this into perspective, the issue that has to be asked is: at what expense to ecological or environmental health is more economic expansion desirable?

Many studies exploring the impact of economic indicators on the environmental quality are rooted with the environmental Kuznets curve (EKC) hypothesis (AlKhars et al. 2022; Dinda 2004; Stern 2017) They have looked at not only how important economic growth is in the EKC modeling framework, but also how important it is in making policies about climate change and sustainability in general. In the study that Apergis (2016) conducted on a panel sample consisting of 15 different economies, they found ambiguous findings. The research conducted by Onafowora and Owoye (2014), which used time series data for eight nations including China, Brazil, Japan, Egypt, Nigeria, Mexico, and South Africa, as well as South Korea, came to a similar conclusion: it was determined that the findings remained inconclusive. South Korea and Japan were discovered to have EKCs that were formed like an inverted U, whereas the other six nations were reported to have EKCs that were shaped like an N. These contradictory findings may be due to variances in the degree of development as well as in the energy mix (renewable versus non-renewable) in each nation.

Narayan and Narayan (2010) conducted another study on 43 emerging economies, and their findings revealed that nations in the Middle East and South Asia are the only ones to have increased environmental quality. A study conducted by Shahbaz et al. (2015) utilizing a time series framework for India discovered that economic expansion plays a substantial effect in the quality of the environment. In contrast, Shahbaz et al. (2018) discovered that a growing economy had a negative impact on the quality of the environment in Japan.

In a research that looked at 27 developed economies, Al-Mulali and Ozturk (2016) came to the conclusion that high levels of economic growth only lead to an improvement in environmental quality over the long run, not over the short term. Using an all-inclusive panel dataset for OECD and developing countries, Özokcu and Özdemir (2017) found proof of an N-shaped (inverted N-shaped) association between growth and environmental deterioration for OECD and emerging nations. This relationship was found to be more prevalent in emerging economies. Due to the fact that the EKC hypothesis was not validated, these findings lead the researchers to propose that increased economic development on its own probably is not enough to improve environmental quality.

Esteve and Tamarit (2012) found, using data from Spain, that the income elasticity between carbon emissions and income is less than one, which implies that the link between the two variables is heading in the direction of diminishing path. According to the findings of Fosten et al. (2012), economic expansion is beneficial to the economy of the UK while also increasing the quality of the environment over the long term. Baek and Kim (2013) for Korea and Tiwari et al. (2013) for India provided support for the EKC hypothesis. On the other hand, the research conducted by Song et al. (2013) on Chinese provinces and Apergis et al. (2017) and Atasoy (2017) on the economy of the USA indicated mixed evidence.

Ang (2007) came to the conclusion that development does not improve the long-term condition of the environment in France owing to the detrimental effect that carbon emissions have on the climate. The evidence for the EKC theory was found to be extremely minimal in the research done by Nasir et al. (2019) on ASEAN. When examining European economies, however, Pham et al. (2020) and Shahbaz et al. (2020a) and Shahbaz et al. (2020b) on the UK and US found substantial support for the EKC assumption. These results indicate substantial variation among nations.

Nevertheless, all of the past study has concentrated on the expansion of the economy rather than the strength of the economy. One definition of economic strength is the capacity of a nation to provide for its own people in terms of material and cultural richness, regardless of the degree of instability present in the external environment (Rim et al. 2020). As a result, this research differentiates itself from other studies that examine economic growth by analyzing the symmetric and asymmetric evidence of the link between economic strength and environmental deterioration.

### Role of research and development in the environment

According to Schumpeter (1942), inventions and innovations introduce “change in technology” into the industrial process. For the innovation process to bear fruit, financial resources must be allocated to research and development (R&D). It is also more probable that the diffusion process will occur if and when inventions and innovations are embraced by individuals, businesses, and governments. Later, in his endogenous growth theory, Romer (1990) maintained that technological development plays a crucial role in the expansion of the economy. As an endogenous variable, technological progress penetrates the manufacturing process during expansion, allowing the market to operate more efficiently.

Weitzman (1997) suggested that in this light, technical development is also very important in reducing pollution. If manufacturers switch to more energy-efficient machinery, an improvement might be seen in environmental quality (Bruyn 1997). Policymakers and governments should thus take into account not only economic growth and financial development but also energy innovation in the industrial process to minimize energy use pollution in their efforts to combat climate change and global warming (Jordaan et al. 2017).

One explanation is the growing significance of the monetary investments needed in energy innovation due to their potential in reducing carbon emissions. A green and sustainable future may be made possible through a low-carbon economy bolstered by energy advances (Anadon et al. 2014; Gallagher et al. 2012). Energy innovation has been recognized as a “pollution internalizing metho” by Fernández et al. (2018) for addressing climate change global warming and fostering long-term sustainable development. When it comes to energy, innovation not only lessens the amount of energy needed for economic activity, but it also lessens the amount of pollution that is produced (Ellabban et al. 2014; Garrone and Grilli 2010). Corporate enterprises are able to devote resources to energy innovation, thanks to government subsidies, which is good for the long-term health of the environment (Chen and Xu 2010; Ockwell et al. 2010).

For instance, businesses may expand operations with less impact on the environment by switching to renewable energy sources (Hall and Bain 2008; Luo et al. 2015). In order to simulate the effects of environmental deterioration using a variety of econometric techniques, many researchers who have looked into environmental concerns have utilized technological developments as a control variable. Yeh and Rubin (2011) examined the connection between climate change and the development of new technologies. According to Jones (1998), cutting down on carbon emissions is possible if more money is put into research and development for new forms of energy. Furthermore, if energy-saving technology is implemented in economic operations, the climate change problem may be addressed at reduced prices (Newell and Pizer 2008). Sohag et al. (2015) found that new technologies have a positive impact on the environment by decreasing carbon emissions and increasing energy efficiency.

Energy-saving technology was also singled out by Smulders and de Nooij (2003) as a beneficial tool for reducing pollution. Parry (2003), on the other hand, stated that improvements in environmental quality due to better pollution management should take precedence over technological advancements. Using Canada as a case study, Jordaan et al. (2017) looked at the potential of energy innovation to cut down on pollution. For the world to meet its greenhouse gas emission goals, they propose that governments and businesses collaborate to accelerate the development of clean energy by allocating more funds to research and development in the energy sector. Energy innovation decreases carbon emissions, as determined by Jin et al. (2017), who studied the connection between energy technology innovation and environmental quality in China. Policy-wise, they advocated that China’s central government should put more money into developing new technologies for the energy sector in order to boost efficiency and lessen the strain on the country’s scarce natural resources.

Using panel data from OECD countries, Ganda (2019) found that both R&D spending and consumption of renewable energy contributed to a more sustainable environment. Other R&D variables, such as the number of researchers and the prevalence of triadic patent families, also positively influenced this outcome. The research team of Alam et al. (2019) used data from G-6 companies to conclude that R&D spending aids in environmental safeguarding. The researchers found evidence in favor of the natural resource-based view’s central claim, namely, that a company may improve its energy efficiency and decrease its carbon intensity by allocating resources and capabilities to activities that have a positive impact on the environment. Investing in renewable energy R&D has little influence on pollution levels, according to a recent research of 19 high-income OECD nations conducted by Koçak and Ulucak (2019). Recent work, while exploring the effect of R&D on carbon emissions in UK, found that the R&D expenditure can reduce the carbon emissions (Shahbaz et al. 2020a, b). Based on the results of these studies, it is essential to explore how do the research and development expenditures affect the environmental deterioration.

## Data and methodology

More than 240 billion metric tons of carbon dioxide equivalent (GtCO2e) have been released into the atmosphere by the 27 member states of the European Union since the beginning of industrialization around 250 years ago. This accounts for around 18% of all global GHG emissions throughout history, becoming top emitter; therefore, this study focuses on the EU-27. The dataset of the article includes panel of EU-27 countries. Table 1 shows all the variables and their relevant proxies. The stock market indices are taken from the specific index of the country as mentioned in Table 7 of the “Appendix”; most of the indices are traded in EUR, exceptions are mentioned in Appendix Table 7. Research and development is used as the percentage of gross domestic product (GDP) of the specific country. Economic growth is measured as the gross domestic product at market prices. Environmental degradation is measured through total of 9 variables: (1) greenhouse gas emissions, measured as tonnes per capita; (2) Particulate matter 2.5 (PM2.5) refers to particles with a diameter of less than 2.5 μm, annual data based on daily averages; (3) particulate matter 10 (PM10) refers to particles with a diameter of less than 10 μm, annual data based on daily averages; (4) carbon monoxide (CO), annual data based on daily averages; (5) sulfur dioxide (SO2), annual data based on daily averages; (6) ozone (O3), based on daily max 8-h averages; (7) nitrogen dioxide (NO2), annual data based on daily averages; (8) observed annual-mean temperature (TEMP); and (9) relative humidity (HUM). The unit of measurement for (2)–(7) is micrograms (one-millionth of a gram) per cubic meter air. Temperature and humidity are measured as degrees Celsius and percentage, respectively. Data frequency is annual for 21 years from 2000 to 2020.

**Table 1:** Variables and proxies

Variable	Proxy	Abbreviation	Data source
Environmental degradation	Greenhouse gases	GHG	EuroStat
	Particulate matter 2.5	PM2.5	European Environmental Agency
	Particulate matter 10	PM10	European Environmental Agency
	Carbon monoxide	CO	European Environmental Agency
	Sulphur dioxide	SO2	European Environmental Agency
	Ozone	O3	European Environmental Agency
	Nitrogen dioxide	NO2	European Environmental Agency
	Temperature	TEMP	Climate Change Knowledge Portal by World Bank
	Humidity	HUM	Climate Change Knowledge Portal by World Bank
Stock market indices	Stock market indices	SK	Relevant Stock Market
Economic strength	Gross domestic product at market price	GDP	EuroStat
Research and development	Percentage of GDP as R&D expenditure	RD	EuroStat

Own elaboration

### Linear panel ARDL

The choice of autoregressive distributed lag (ARDL) is due to the reason that irrespective of the integrated order, i.e., *I(0)* or *I(1)*, ARDL is applicable (Akmal 2007).^1^ This study follows Salisu and Isah (2017)’s methodological approach to evaluate the nexus. First, it is assumed that environmental degradation will react equally to increases and decreases in the stock market indices, economic growth, and research and development. Subsequently, the assumption is removed so that it may account for both types of movements in the analysis. Therefore, the symmetric form of the panel ARDL of the model is shown as:

1 $$\begin{array}{l}{\Delta g}_{\mathrm{nit}}=\exists_{0\mathrm i}+\exists_{1\mathrm i}g_{\mathrm i,\mathrm t-1}+\exists_{2\mathrm i}p_{\mathrm i,\mathrm t-1}+\exists_{3\mathrm i}e_{\mathrm t-1}+\exists_{4\mathrm i}r_{\mathrm i,\mathrm t-1}+\Sigma_{j=1}^{N1}\lambda_{\mathrm{ij}}{\Delta g}_{\mathrm i,\mathrm t-\mathrm j}+\Sigma_{j=1}^{N1}\gamma_{\mathrm{ij}}{\Delta p}_{\mathrm t-\mathrm j}+\Sigma_{j=1}^{N1}\vartheta_{ij}{\Delta e}_{t-j}+\Sigma_{j=1}^{N1}\psi_{\mathrm{ij}}{\Delta r}_{\mathrm t-\mathrm j}+\mu_i+\varepsilon_{\mathrm{it}}\\i=1,2,\dots,N;\;t=1,2,\dots,N\end{array}$$

Here, ${g}_{\mathrm{nit}}$ is the log of the nth environmental variable; ${p}_{\mathrm{it}}$ denotes log of stock price index; ${e}_{\mathrm{it}}$ is the log of gross domestic product at market price; ${r}_{\mathrm{it}}$ is the log of research and development expenditure; ${\mu }_{\mathrm{i}}$ is the group-specific effect; *i* is the country; and *t* is time period.

Assuming that ${\Delta g}_{\mathrm{ni},\mathrm{t}-\mathrm{j}},{\Delta p}_{\mathrm{t}-\mathrm{j }}, {\Delta e}_{\mathrm{t}-\mathrm{j} }, {\Delta r}_{\mathrm{t}-\mathrm{j} }=0$, the long-run slope (elasticity) coefficient is calculated for each cross-section as $-\frac{{\exists }_{\mathrm{Ni}}}{{\exists }_{1\mathrm{i}}}$. Accordingly, the short-term forecast for stock market indices, GDP growth, and R&D spending is calculated to be ${\gamma }_{\mathrm{ij}}, {\vartheta }_{\mathrm{ij}},$ and ${\psi }_{\mathrm{ij}}$. Equation (1) may be rewritten to incorporate an error correcting term in the following format:

2 $${\Delta g}_{\mathrm{nit}}= {\delta }_{\mathrm{i}},{\upsilon }_{\mathrm{i},\mathrm{t}-1}+ {\Sigma }_{j=1}^{N1}{\lambda }_{\mathrm{ij}}{\Delta g}_{\mathrm{i},\mathrm{t}-\mathrm{j} }+{\Sigma }_{j=1}^{N1}{\gamma }_{\mathrm{ij}}{\Delta p}_{\mathrm{t}-\mathrm{j} }+{\Sigma }_{j=1}^{N1}{\vartheta }_{\mathrm{ij}}{\Delta e}_{\mathrm{t}-\mathrm{j} }+{\Sigma }_{j=1}^{N1}{\psi }_{\mathrm{ij}}{\Delta r}_{\mathrm{t}-\mathrm{j }}+{\mu }_{\mathrm{i}}+ {\varepsilon }_{\mathrm{it}}$$

where ${\upsilon }_{\mathrm{i},\mathrm{t}-1}= {g}_{\mathrm{i},\mathrm{t}-1}-{\phi }_{0\mathrm{i}}- {\phi }_{1\mathrm{i}}{p}_{\mathrm{t}-1}-{\phi }_{2\mathrm{i}}{e}_{\mathrm{t}-1}- {\phi }_{3\mathrm{i}}{r}_{\mathrm{t}-1}$ is the linear error correction term for each unit; the parameter ${\delta }_{\mathrm{i}}$ is the error-correcting speed of adjustment term for each unit which is also equivalent to ${\exists }_{1\mathrm{i}}$. The parameters ${\phi }_{ni}$ are computed as $-\frac{{\exists }_{\mathrm{Ni}}}{{\exists }_{1\mathrm{i}}}$. It can be seen that in Eqs. (1) and (2), there are no decompositions of stock indices, GDP, and R&D into positive and negative changes; hence, the assumption of symmetric impact of stock indices, GDP, and R&D shocks on environmental degradation under this scenario.

### Non-linear panel ARDL

This variation of the panel ARDL, known as the nonlinear panel ARDL, allows for an asymmetric response of environmental degradation to stock indices, GDP, and R&D, unlike the symmetric case. Positive and negative shocks are not predicted to have the same effect on environmental degradation under this scenario. Thus, the asymmetric form of Eq. (1) is represented as follows:

3 $${\Delta g}_{\mathrm{nit}}= {\exists }_{0\mathrm{i}}+{\exists }_{1\mathrm{i}}{g}_{\mathrm{i},\mathrm{t}-1}+{\exists }_{2\mathrm{i}}^{+}{p}_{\mathrm{t}-1}^{+}+{\exists }_{2\mathrm{i}}^{-}{p}_{\mathrm{t}-1}^{-}+{\exists }_{3\mathrm{i}}^{+}{e}_{\mathrm{t}-1}^{+}+{\exists }_{3\mathrm{i}}^{-}{e}_{\mathrm{t}-1}^{-}+{\exists }_{4\mathrm{i}}^{+}{r}_{\mathrm{t}-1}^{+}+{\exists }_{4\mathrm{i}}^{-}{r}_{\mathrm{t}-1}^{-}+ {\Sigma }_{\mathrm{j}=1}^{N1}{\lambda }_{\mathrm{ij}}{\Delta g}_{\mathrm{i},\mathrm{t}-\mathrm{j} }+{\Sigma }_{\mathrm{j}=1}^{N2}({{\gamma }_{\mathrm{ij}}^{+}{p}_{\mathrm{t}-1}^{+}+{\gamma }_{\mathrm{ij}}^{-}{p}_{\mathrm{t}-1}^{-})}+{\Sigma }_{\mathrm{j}=1}^{N3}({{\vartheta }_{\mathrm{ij}}^{+}{e}_{\mathrm{t}-1}^{+}+{\vartheta }_{\mathrm{ij}}^{-}{e}_{\mathrm{t}-1}^{-})}+{\Sigma }_{\mathrm{j}=1}^{N3}({{\psi }_{\mathrm{ij}}^{+}{r}_{\mathrm{t}-1}^{+}+{\psi }_{\mathrm{ij}}^{-}{r}_{\mathrm{t}-1}^{-})}+{\mu }_{\mathrm{i}}+ {\varepsilon }_{\mathrm{it}}$$

where ${p}_{\mathrm{t}}^{+}$, ${p}_{\mathrm{t}}^{-}$*,* ${e}_{\mathrm{t}}^{+}$, ${e}_{\mathrm{t}}^{-}$*,* ${r}_{\mathrm{t}}^{+},$ and ${r}_{\mathrm{t}}^{-}$ denote the positive and negative shocks of stock indices, GDP, and R&D, respectively. The long-run (elasticity) coefficients for ${p}_{\mathrm{t}}^{+}$, ${p}_{\mathrm{t}}^{-}$*,* ${e}_{\mathrm{t}}^{+}$, ${e}_{\mathrm{t}}^{-}$*,* ${r}_{\mathrm{t}}^{+},$ and ${r}_{\mathrm{t}}^{-}$ are calculated as $-\frac{{\exists }_{\mathrm{Ni}}^{+}}{{\exists }_{1\mathrm{i}}}$ and $-\frac{{\exists }_{\mathrm{Ni}}^{-}}{{\exists }_{1\mathrm{i}}}$. As outlined below, these shocks are calculated as positive and negative partial sum decompositions of stock market indices, gross domestic product (GDP), and research and development (R&D).

$$\begin{array}{c}{p}_{t}^{+}={\Sigma }_{k=1}^{t}{\Delta p}_{ik}^{+}={\Sigma }_{k=1}^{t}\mathrm{max}({\Delta p}_{ik},0)\\ {p}_{t}^{-}={\Sigma }_{k=1}^{t}{\Delta p}_{ik}^{-}={\Sigma }_{k=1}^{t}\mathrm{min}({\Delta p}_{ik},0)\end{array}$$

$$\begin{array}{c}{e}_{t}^{+}={\Sigma }_{k=1}^{t}{\Delta e}_{ik}^{+}={\Sigma }_{k=1}^{t}\mathrm{max}({\Delta p}_{ik},0)\\ {e}_{t}^{-}={\Sigma }_{k=1}^{t}{\Delta e}_{ik}^{-}={\Sigma }_{k=1}^{t}\mathrm{min}({\Delta p}_{ik},0)\end{array}$$

$$\begin{array}{c}{r}_{t}^{+}={\Sigma }_{k=1}^{t}{\Delta r}_{ik}^{+}={\Sigma }_{k=1}^{t}\mathrm{max}({\Delta p}_{ik},0)\\ {r}_{t}^{-}={\Sigma }_{k=1}^{t}{\Delta r}_{ik}^{-}={\Sigma }_{k=1}^{t}\mathrm{min}({\Delta p}_{ik},0)\end{array}$$

The error correction version of Eq. (3) yields the following:

4 $${\Delta g}_{nit}= {\tau }_{i}{\xi }_{i,t-1}+ {\Sigma }_{j=1}^{N1}{\lambda }_{ij}{\Delta g}_{i,t-j }+{\Sigma }_{j=1}^{N2}({{\gamma }_{ij}^{+}{p}_{t-1}^{+}+{\gamma }_{ij}^{-}{p}_{t-1}^{-})}+{\Sigma }_{j=1}^{N3}({{\vartheta }_{ij}^{+}{e}_{t-1}^{+}+{\vartheta }_{ij}^{-}{e}_{t-1}^{-})}+{\Sigma }_{j=1}^{N3}({{\psi }_{ij}^{+}{r}_{t-1}^{+}+{\psi }_{ij}^{-}{r}_{t-1}^{-})}+{\mu }_{i}+ {\varepsilon }_{it}$$

In the asymmetric panel ARDL provided in Eq. (4), ${\tau }_{\mathrm{i}}$ is the coefficient of error correction term that quantifies how long it takes the system to converge to its long-run equilibrium in the presence of a shock, and the error-correction term ${\xi }_{\mathrm{i},\mathrm{t}-1}$ captures the long-run equilibrium.

## Preliminary testing

Table 2 depicts the descriptive statistics of the sample. The average stock market index in the EU-27 is 6108.65. GDP at market price is on average 243,571.31 Euros and the average expenditure on research and development in EU-27 is 1.47% of the GDP. On average there is emission of 9.24 tonnes per capita in EU-27. The particulate matters 2.5 and 10 in the air is at 15.52 and 27.10 µg/m3 on average, respectively. CO is on 0.47 µg/m3 on average with standard deviation of 0.29 µg/m3, SO2 has the mean value of 5.57 µg/m3, O3 average value is 53.16 µg/m3, and average value for NO2 is at 22.25 µg/m3. The average temperature in EU-27 is 10.63 °C with the deviation of 3.90°. Average relative humidity is at 75.68%. The dataset is not normally distributed in its original form apart from temperature. All the variables are positively skewed, apart from R&D expenses and Humidity.

**Table 2:** Descriptive statistics

Variables	Obs	Mean	Std. Dev	p1	p99	Skew	Kurt
SK	534	6108.65	10,451.27	49.87	54,558.15	3.12	13.34
GDP	565	24,357.31	16,526.57	4170.00	83,550.00	1.52	6.05
RD	557	1.47	0.89	0.32	3.54	0.74	2.48
GHG	567	9.24	4.65	1.60	26.60	1.33	6.10
PM2.5	444	15.52	6.72	4.69	32.77	2.06	15.82
PM10	540	27.10	8.95	11.53	50.92	0.68	3.23
CO	520	0.47	0.29	0.00	1.62	2.90	15.45
SO2	523	5.57	4.67	0.58	23.96	2.23	10.31
O3	523	53.16	8.77	34.87	76.80	0.83	6.23
NO2	550	22.25	7.18	6.97	42.38	0.58	4.21
TEMP	567	10.63	3.90	2.19	20.06	0.35	3.50
HUM	567	75.68	4.88	63.51	83.61	-0.63	2.72

Own elaboration

**Fig. 1:**
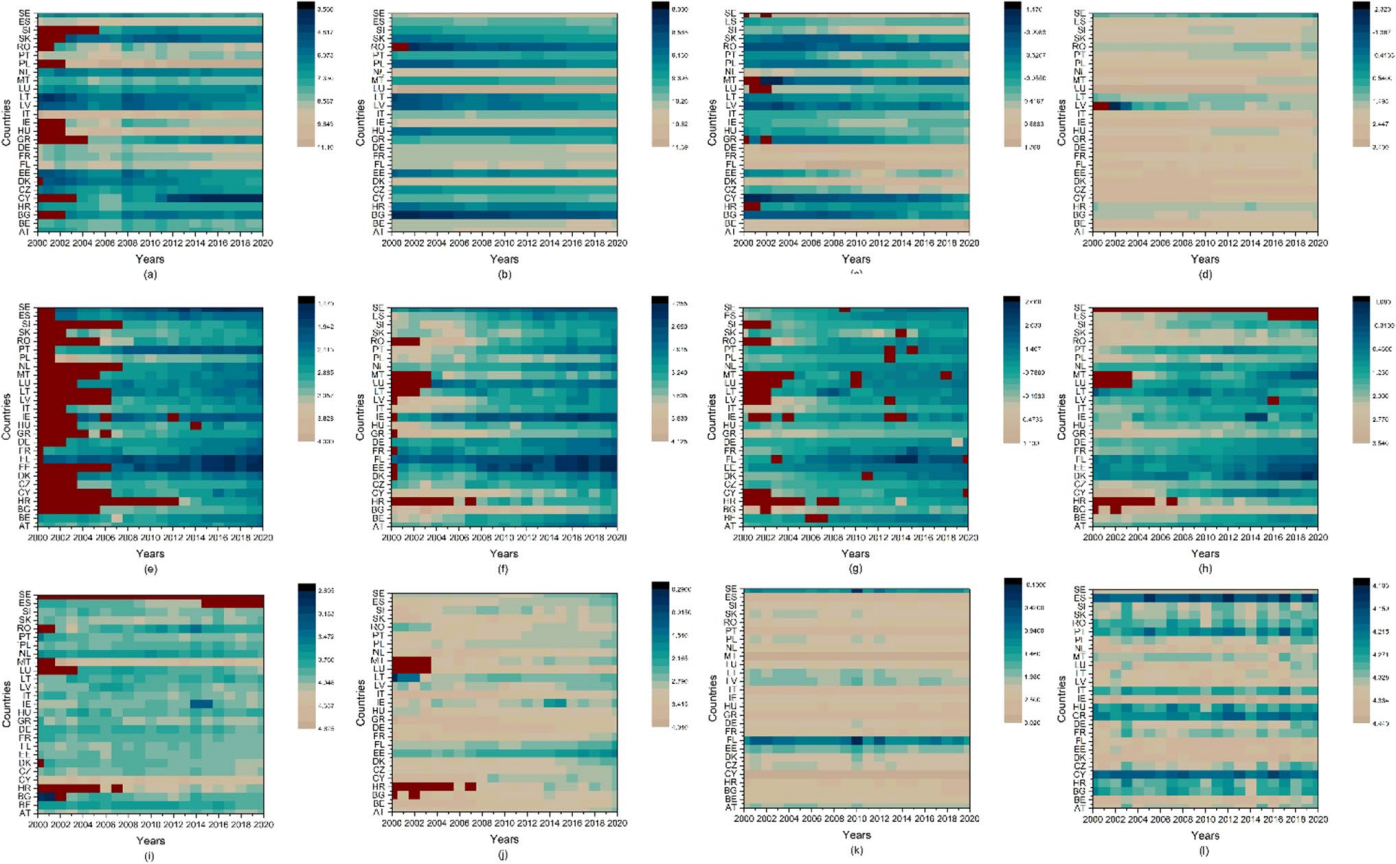
Trend of variables. Source: own elaboration

To explore trends in variables, Fig. 1 shows the contour maps of the variables in the natural logged form. The stock market indices (Fig. 1a) mostly remain in average index value from 4.502 to 7.330. However, few higher value can be observed such as for Poland, Italy, and Hungary. Countries such as Denmark, Luxembourg, and the Netherlands have higher economic strength (Fig. 1b) as compared to the rest of the sample. Denmark spends a larger chunk of GDP to R&D (Fig. 1c) in the sample alongside Austria, Belgium, Finland, France, and Germany. The greenhouse gases (Fig. 1d) remain on the higher side, only Latvia has lower GHG emissions. Particulate matter at a density of 2.5 μg (one-millionth of a gram) per cubic meter air as shown in Fig. 1e is at a higher level for Poland, Greece, and Bulgaria and at a significantly lower level for Ireland, Estonia, Finland, and Sweden. Data for PM2.5 for some countries start after 2002, and in the extreme case of Croatia, it starts after 2012. PM10 (Fig. 1f) is significantly high for almost all the countries in EU-27 from year 2000 to 2006 then a decline is visible; however, for Bulgaria and Greece, it continues to be high until 2018; similar to PM2.5, PM10 is at much lower level for Ireland and Finland. Carbon monoxide presence in environment is depicted in Fig. 1g. The natural logged value of CO remains in the negative due to the reason that the original values of CO are under 1, there are no specific visible trends; however, the CO are low for Finland and Belgium. Sulfur dioxide (SO2) (Fig. 1h) is at a higher level for Bulgaria, Romania, and Slovakia and lower level for Finland, Estonia, and Denmark; complete data for Sweden and recent years for Spain is not available. Ozone (O3) (Fig. 1i) is at higher for Malta, Cyprus, and Croatia, and similar to SO2 the data is not available for Sweden and Spain. NO2 (Fig. 1j) levels of Estonia, Finland, and Latvia are at lower levels than the rest of the sample. Temperature also remains lower Finland, Estonia, and Sweden, for temperature change in each year is more important than the level of temperature; however, there is no trend of significant increase or decrease is available. Relative humidity (HUM) is lower for Spain, Greece, and Cyprus for the rest of sample; there is one wave for years 2013 and 2014 which increases the humidity for given year.

The results of the unit root test are depicted in Table 3. Null statistical hypothesis is that all the panels are non-stationary. Most of variables are integrated at order one (I (1)); however, the SK, CO, O3, and humidity are integrated at order zero (I (0)).

**Table 3:** Unit root test for stationarity

	lnSK	lnGDP	lnRD	lnGHG	lnPM2.5	lnPM10	lnCO	lnSO2	lnO3	lnNO2	lnTEMP	lnHUM
Augmented Ducky Fuller I (0)	113.34***	72.41**	59.7	28.99	42.61	23.81	150.22***	37.43	118.78***	43.6	62.1	237.34***
Augmented Ducky Fuller I (1)	394.17 ***	129.17***	178.53***	201.75***	360.94***	432.49***	289.71***	312.74***	409.80***	219.43***	557.06***	609.54***
Im-Pesaran-Shin I (0)	− 3.81***	− 2.02**	2.71	4.79	2.55	3.55	− 3.07**	2.08	− 3.18***	5.68	− 1.99**	− 8.86***
Im-Pesaran-Shin I (1)	− 13.78***	− 5.32***	− 6.96***	− 6.84***	− 12.15***	− 14.60***	− 10.27***	− 11.18***	− 14.35***	− 7.46***	− 17.74***	− 19.30***
Perasan CADF I (0)	1.5	− 1.84***	− 1.59*	− 1.34	− 2.39***	− 2.132	− 3.14***	− 2.40***	− 2.12**	− 0.31	− 2.338	− 2.75***
Perasan CADF I (1)	− 5.97***	− 2.29**	-4.48***	− 3.42***	− 5.54***	− 6.68***	− 7.15***	− 3.04***	− 8.57***	− 5.38***	− 4.33***	− 4.17***
Perasan CD	36.49***	57.77***	34.85***	34.31***	47.53***	59.77***	41.01***	48.24***	11.43***	38.29***	49.36***	19.96***
No of cross-sections	27	27	27	27	27	27	27	26	26	27	27	27

^***^, **, and * represent the level of significance at 1%, 5%, and 10%, respectively

Own elaboration

In addition, all the variables have Perasan cross-sectional dependence test significant at 1%. This suggests that the cross-sections are based on a heterogeneous structure. As a result, the fundamental framework for estimate presented in this study, which takes into account the underlying heterogeneity and non-stationarity in the panel data series, is appropriate for the analyses that this study follows. In summary, the findings of the unit root test provide additional support for the suitability of selecting the panel-ARDL model as the best estimate framework within the scope of this investigation.

## Symmetric and asymmetric behavior

After estimating both of the equations using mean group and pooled mean group method, we next put the results of these estimation methods through the Hausman test. The pooled mean group estimator is assumed to be used when the null hypothesis is not rejected, but the mean group estimator is assumed to be used when the null hypothesis is rejected. To put it another way, the pooled mean group estimator is the efficient estimator when the null hypothesis is being considered, and the mean group estimator is the efficient estimator when the alternative hypothesis is being considered. Hausman test findings provide strong support for the pooled mean group estimator as the most effective estimator when it comes to modeling nexus between environmental degradation, stock market indices, economic strength, and R&D expenditure.

Table 4 presents the results of symmetric evidence of the nexus. The stock market indices are irrelevant for greenhouse gases, particulate matter 2.5, particulate matter 10, carbon monoxide, and nitrogen dioxide in the long run. Impact of stock markets on ozone, temperature, and humidity is significant and negative in the long run; impact on temperature is significant and positive. Stock markets in the short run show increasing significant impact for all variables except for greenhouse gases and humidity, which do not get affected by the stock markets. The effect which is negative in the long run for SO2 and O3 becomes positive.

**Table 4:** Symmetric impact on environmental degradation (*g)*

	GHG		PM2.5		PM10		CO		SO2		O3		NO2		TEMP		HUM	
	Coef	Std Err	Coef	Std Err	Coef	Std Err	Coef	Std Err	Coef	Std Err	Coef	Std Err	Coef	Std Err	Coef	Std Err	Coef	Std Err
$p$	0.007	0.012	-0.018	0.015	0.007	0.006	0.016	0.018	− 0.012***	0.018	− 0.046***	0.008	− 0.091	0.029	0.010***	0.005	− 0.006***	0.003
$e$	0.283***	0.073	0.352***	0.096	0.048	0.036	− 1.474***	0.139	− 0.714***	0.133	0.204	0.043	− 0.350***	1.264	0.012	0.035	− 0.016	0.014
$r$	− 0.106***	0.038	0.194***	0.023	0.010	0.012	0.263***	0.057	− 0.090	0.037	− 0.021	0.014	1.749**	1.866	− 0.019**	0.009	0.007	0.007
$\Delta p$	− 0.009	0.018	0.036**	0.015	0.022***	0.005	0.048***	0.010	0.036***	0.007	0.042***	0.004	0.116***	0.021	-0.062***	0.014	− 0.003	0.004
$\Delta e$	0.966***	0.198	0.443***	0.112	0.465***	0.069	0.544***	0.095	0.193	0.081	0.027***	0.045	0.704**	0.128	− 0.017	0.116	0.081**	0.034
$\Delta r$	− 0.028	0.104	− 0.081	0.115	0.020	0.045	− 0.141	0.102	0.067	0.075	0.038**	0.037	0.118	0.050	0.134	0.105	− 0.010	0.023
${\upsilon }_{i,t-1}$	− 0.554***	0.058	− 0.698***	0.054	− 1.229***	0.021	− 0.584***	0.039	− 0.630***	0.033	− 0.984***	0.026	− 0.949***	0.076	− 1.061***	0.046	− 1.013***	0.055
Constant	− 0.264***	0.047	− 0.161***	0.054	3.751***	0.069	8.013***	0.541	5.700***	0.320	2.247***	0.063	− 4.096***	1.665	2.162***	0.158	4.590***	0.242
PMG stats																		
No. of observations		495		497		497		497		497		497		497		497		497
No. of cross-sections		27		27		27		27		27		27		27		27		27
Log likelihood		886.15		726.64		1009.04		18.40		806.75		1060.42		945.65		891.04		1328.04
Hausman		1.87		4.03		1.28		1.6		3.59		5.67		7.29		3.15		3.09
${\chi }^{2}$		− 0.5991		0.2580		0.7342		0.6584		0.3097		0.1286		0.0633		0.3690		0.3773

^***^, **, and * represent the level of significance at 1%, 5%, and 10%, respectively

Own elaboration

Economic strength is very much relevant both in the long run and short run. The long run estimates show that effect of economic strength on the greenhouse gases, particulate matter 2.5, and nitrogen dioxide in air is significant and positive. This effect is significant but negative for CO and SO2. In the short run, apart from model with temperature and SO2 as the dependent variable, all other models show that the economic strength increases the environmental degradation. Nevertheless, the magnitude of effect in the short run is larger than that in the long run.

The research and development expenditure causes an increasing significant impact on PM2.5, CO, and NO2 in the long run and a decreasing effect can be seen for temperature and greenhouse gases, but in short run, it does not cause much effect on the environmental degradation except for O3 which shows a positive significant effect from R&D. The existence of only long-run impact is due to the fact that the research and development is a prolonged process, and the effects are visible over a longer period of time.

The results of the asymmetric nexus are presented in Table 5, Hausman test supports the PMG estimator. The stock market indices have become significant as contrasted to the symmetric estimation, the long-run positive value of stock market indices creates a statistically significant increase in GHG, PM2.5, PM10, and NO2; however, a statistically significant decrease is observed for CO, O3, and temperature. For the short-run effect of positive stock market indices, values show that the stock market indices have a negative significant relationship with greenhouse gases, PM2.5, and PM10, whereas a positive significant relationship with CO and O3. Negative stock market indices’ values show that in the long run, it has a negative significant relationship with GHG, PM2.5, O3, and NO2 and a positive significant relationship with PM10, CO, and SO2. In the short run, the effect of negative stock market index values has a negative significant relationship with GHG and TEMP. PM10, O3, and NO2 are increased as the stock market indices show a bearish behavior.

**Table 5:** Asymmetric impact on greenhouse gases (*g)*

	GHG		PM2.5^i^		PM10 ^i^		CO ^i^		SO2^1^		O3 ^i^		NO2 ^i^		TEMP ^i^		HUM ^i^	
	Coef	Std Err	Coef	Std Err	Coef	Std Err	Coef	Std Err	Coef	Std Err	Coef	Std Err	Coef	Std Err	Coef	Std Err	Coef	Std Err
${p}^{+}$	0.094***	0.02	0.260***	0.024	0.040***	0.007	− 0.080**	0.035	− 0.043	0.031	− 0.042***	0.010	0.067***	0.014	− 0.022***	0.006	− 0.128	0.123
${p}^{-}$	− 0.048***	0.014	− 0.035**	0.018	0.013**	0.007	0.232***	0.032	0.324***	0.038	− 0.028***	0.008	− 0.124***	0.013	− 0.006	0.005	0.028	0.036
${e}^{+}$	1.415***	0.183	0.638***	0.168	0.155***	0.049	− 0.856***	0.251	− 0.267	0.209	0.373***	0.065	0.927***	0.123	− 0.072	0.057	0.567	0.738
${e}^{-}$	1.203***	0.273	− 0.995***	0.221	-0.084	0.076	− 0.276	0.391	0.258	0.365	0.269***	0.090	1.042***	0.237	0.379***	0.086	− 0.511	0.509
${r}^{+}$	0.190***	0.074	0.262***	0.059	− 0.020	0.016	− 0.031	0.089	0.036	0.081	0.001	0.022	0.120***	0.036	− 0.025**	0.011	0.053	0.182
${r}^{-}$	− 0.325***	0.08	− 0.393***	0.085	0.074***	0.024	0.029	0.121	0.068	0.114	0.012	0.032	− 0.173***	0.062	0.080***	0.028	− 0.358	0.386
$\Delta {p}^{+}$	− 0.077*	0.046	− 0.094 ***	0.024	− 0.029*	0.015	0.141***	0.035	0.035	0.094	0.053***	0.013	0.006	0.018	0.018	0.018	− 0.012	0.026
${\Delta p}^{-}$	0.084***	0.029	0.000	0.015	0.016*	0.009	0.020	0.019	− 0.098	0.064	0.048***	0.010	0.101***	0.013	− 0.070***	0.024	0.007	0.021
$\Delta {e}^{+}$	− 0.305	0.306	− 1.296***	0.293	− 0.414***	0.121	− 0.834**	0.337	− 0.499	0.618	− 0.629***	0.146	− 0.873***	0.158	0.494***	0.160	0.274**	0.169
${\Delta e}^{-}$	0.500**	0.249	1.978***	0.343	0.658***	0.146	0.958***	0.253	− 0.751	0.887	0.265**	0.124	1.231***	0.279	− 0.565	0.406	0.362	0.202
${\Delta r}^{+}$	− 0.11	0.196	− 0.383***	0.127	− 0.054	0.096	− 0.079	0.172	− 0.269	0.313	0.018	0.075	− 0.297***	0.097	0.844	0.647	− 0.114	0.117
${\Delta r}^{-}$	0.133	0.158	0.470	0.383	0.012	0.172	− 0.073	0.159	− 1.051*	0.548	-0.072	0.132	0.371*	0.198	− 0.177***	0.173	− 0.034***	0.193
${\xi }_{t-1}$	− 0.504***	0.064	− 0.748***	0.043	− 1.223***	0.041	− 0.469***	0.047	− 0.617***	0.100	− 0.980***	0.051	− 0.622***	0.062	− 1.107***	0.037	− 1.509***	0.079
Constant	1.125***	0.169	2.119***	0.124	4.369***	0.146	− 0.163***	0.014	1.419***	0.288	3.842***	0.201	2.007***	0.197	2.450	0.140	6.549	0.343
PMG Stats																		
No of observations		538		540		540		540		494		520		540		540		540
No of cross sections		27		27		27		27		26		26		27		27		27
Log likelihood		#####		894.00		1210.81		847.48		443.30		1185.99		1097.33		1106.79		1391.12
Hausman Test		0.52		0.96		2.9		2.2		-		4.77		0.72		0.33		11.16
${\chi }^{2}$		0.998		0.9869		0.8219		0.9004		-		0.5738		0.994		0.9993		0.0836

^***^, **, and * represent the level of significance at 1%, 5%, and 10%, respectively

“i,” dependent variables are interpolated for missing values due to the restriction of model

“1,” PMG model couldn’t run due to model restrictions

Own elaboration

Either positive or negative economic strength both prompt the environmental degradation in the long run; however, the magnitude of effect is larger for the positive economic strength. Long-run estimates of positive economic strength only cause a statistically significant negative impact for CO and negative economic strength causes decrease in PM2.5. In the short run, the behavior is a bit different as positive economic strength has significant positive impact on TEMP and HUM and negative impact on PM2.5, PM10, CO, O3, and NO2. The negative economic strength has an increasing effect in all the significant dependent variables.

Positive research and development expenses in the long run increase GHG, PM2.5, and NO2 while decrease temperature. The negative research and development expenses in the long run decrease GHG, PM2.5, and NO2 and increase PM10 and TEMP. Short-run effect of positive and negative research and development expenses is negative, as positive R&D decreases PM 2.5 and NO2 and negative R&D decreases temperature and humidity. Our work contrasts with the evidence provided by Zeqiraj et al. (2020).

Table 6 shows the Wald test for testing the existence of asymmetries in the long and short run. The null statistical hypothesis for the relationship is that the positive and negative shocks are alike. For stock market growth, the asymmetry exists in the long run in all variables except for O3, and in the short run, the asymmetry is not validated for SO2 and O3. Economic growth tends to have asymmetric behavior in the context of PM2.5, PM10, TEMP, and HUM in the long run; however, in the short run, the asymmetry is evident expect for SO2. Research and development is mostly symmetric in the short run as it only shows asymmetric behavior when tested impact on PM2.5. Asymmetry is more obvious in the long run, as it is validated for GHG, PM2.5, PM10, NO2, TEMP, and HUM.

**Table 6:** WALD test for verification of presence of asymmetry

		GHG	PM2.5	PM10	CO	SO2	O3	NO2	TEMP	HUM
$p$	χ2	31.83***	87.790***	5.780**	37.890***	45.410***	0.930	89.280***	3.990**	72.720***
	p	0.000	0.000	0.016	0.000	0.000	0.334	0.000	0.046	0.000
$e$	χ2	0.31	25.420***	5.060**	1.220	1.020	0.640	0.150	13.270***	58.000***
	p	0.575	0.000	0.024	0.270	0.312	0.423	0.697	0.000	0.000
$r$	χ2	21.29***	28.760***	7.960***	0.130	0.040	0.060	14.110***	9.700***	0.000
	p	0.000	0.000	0.005	0.714	0.837	0.813	0.000	0.002	0.969
$\Delta p$	χ2	9.07***	9.720***	4.540**	8.680***	0.950	0.060	11.960***	6.460**	43.070***
	p	0.003	0.002	0.033	0.003	0.329	0.809	0.001	0.011	0.000
$\Delta e$	χ2	3.10*	38.470***	21.800***	13.080***	0.070	17.310***	43.260***	6.110**	14.930***
	p	0.078	0.000	0.000	0.000	0.790	0.000	0.000	0.013	0.000
$\Delta r$	χ2	1.100	3.640**	0.080	0.000	1.600	0.250	8.360***	1.760	0.590
	p	0.294	0.057	0.783	0.982	0.206	0.616	0.004	0.185	0.444

^***^, **, and * represent the level of significance at 1%, 5%, and 10%, respectively

Own elaboration

### Spatial and temporal graphs

**Fig. 2:**
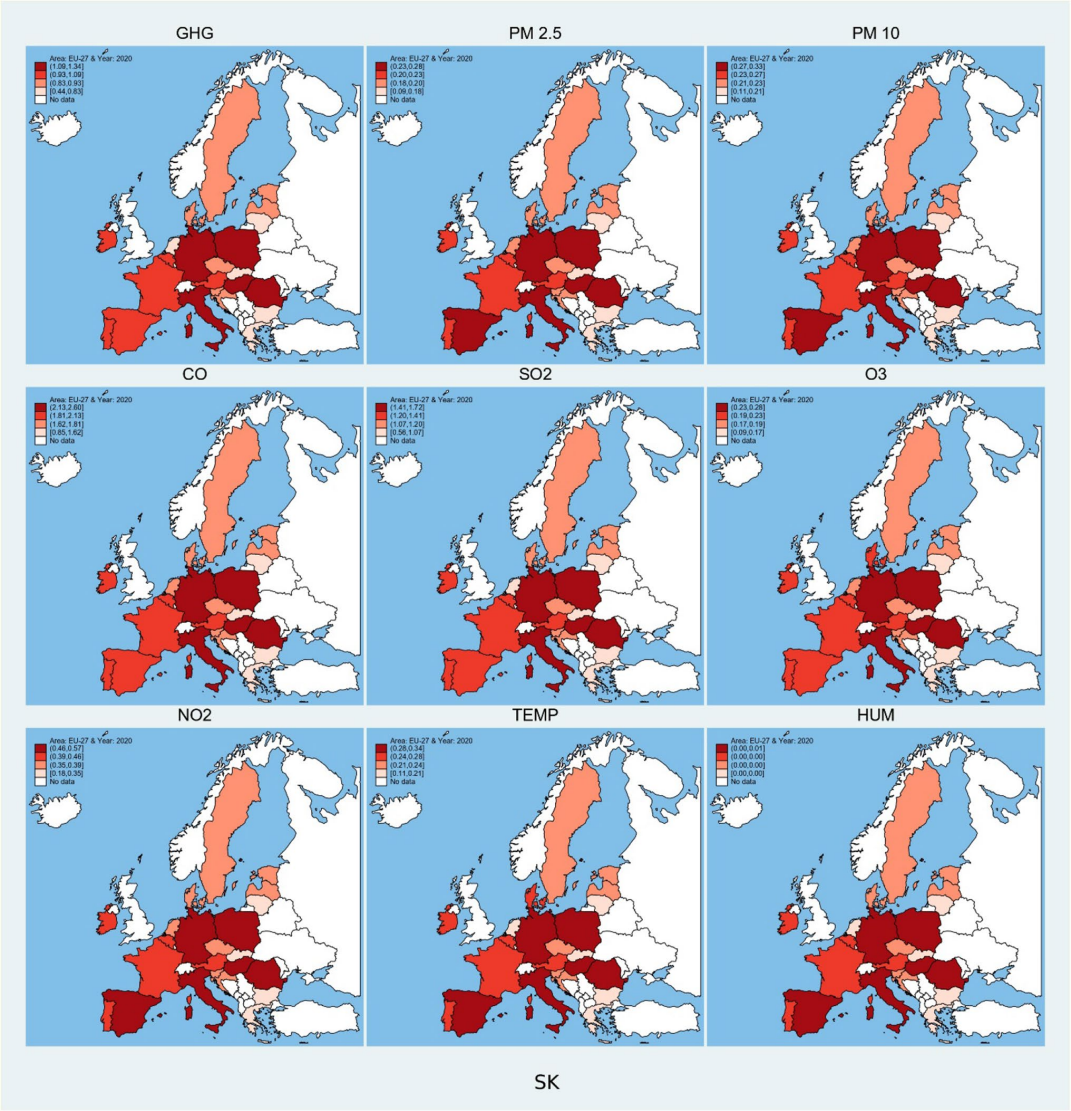
Spatial plot of predicted values of environmental degradation based on influence of stock market indices. (Figs. 2, 3, and 4 are in absolute values to present the spatial trend). Source: own elaboration

**Fig. 3:**
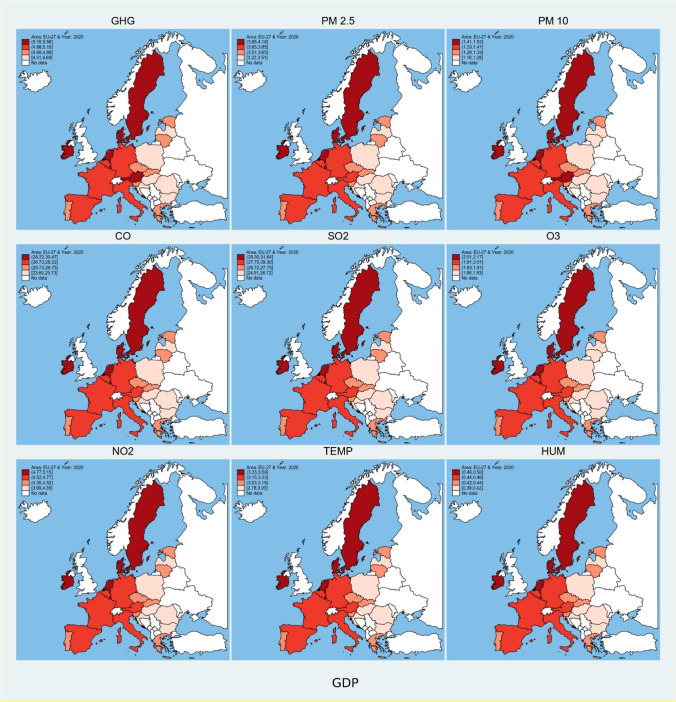
Spatial plot of predicted values of environmental degradation based on influence of economic strength. Source: own elaboration

**Fig. 4:**
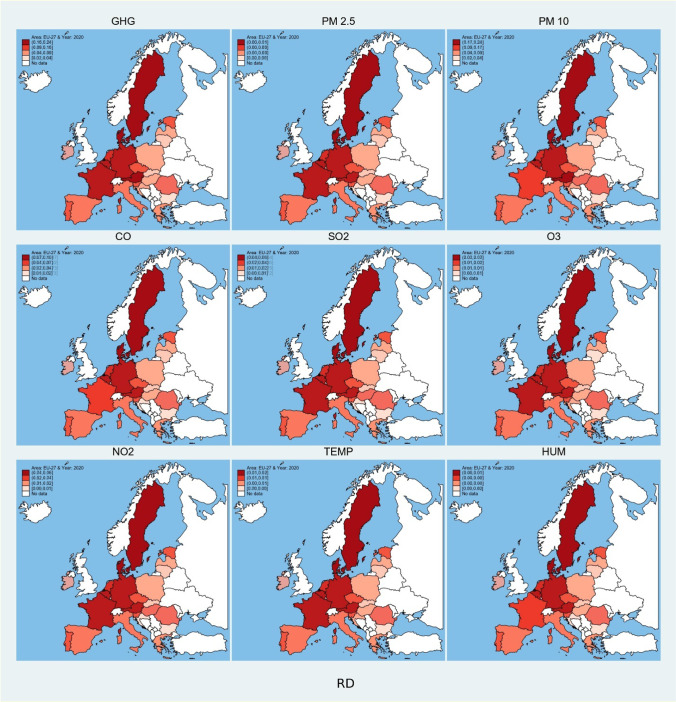
Spatial plot of predicted values of environmental degradation based on influence of research and development expenses. Source: own elaboration

**Fig. 5:**
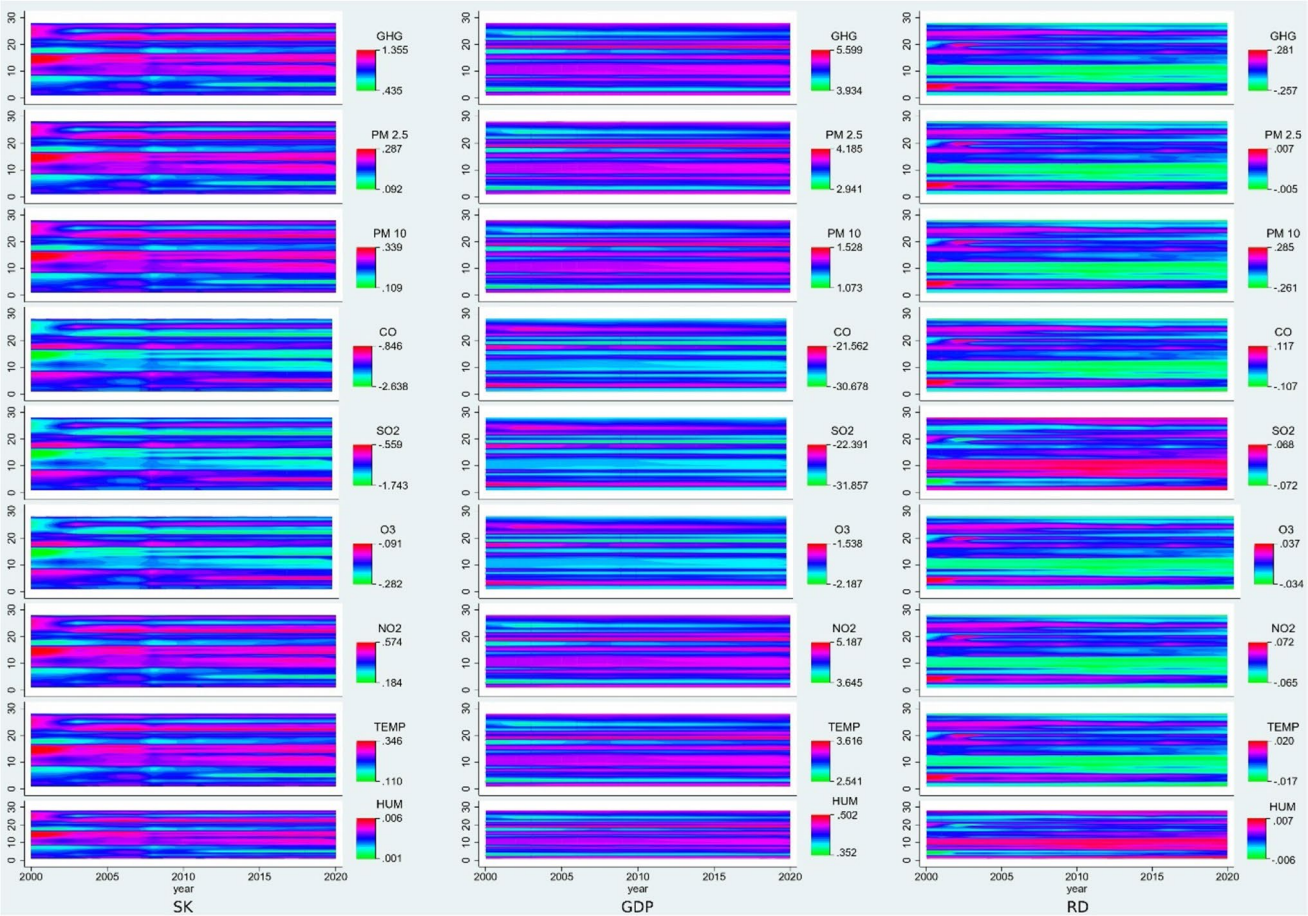
Contour temporal plots of predicted values of environmental degradation based on influence of stock market indices, economic strength, and research and development expenses. (Countries are plotted on y-axis of all the individual contour plots in reverse alphabetical order, i.e., 1 = AT, 2 = BE, …,27 = ES), *Source: own elaboration*

In addition to the results presented in Tables 4 and 5, this study provides with the spatial and temporal graphs. Figures 2, 3, 4, and 5 show the predicted value of dependent variable using autoregressive distributed lag model; all three independent variables are tested individually to gauge individual effect on the environmental variables. The spatial graphs have four groups based on the effect size and the groups remain almost similar. The stock market indices (Fig. 2) contribute the most towards environmental degradation in Poland, Germany, Italy, Hungary, Romania, and Spain. The least effect caused by the stock markets is in the Netherlands, Luxembourg, Greece, Slovakia, and Lithuania. Considering Fig. 5, the temporal aspect of the predicted values shows that as the contribution of stock market indices towards the environmental degradation increases over time. Cyprus, Greece, Ireland, and Portugal have a converging trend towards the lower effect, whereas in Germany, Poland, Hungary, Estonia, Finland, the Netherlands, Latvia, Lithuania, and Sweden, the effect of stock market indices increases the environmental degradation.

Economic strength (Fig. 3) has the most contribution in increasing environmental degradation in Finland, Slovenia, the Netherlands, and Ireland, and least in Poland, Romania, Hungary, Bulgaria, Croatia, and Lithuania. Figure 5 shows that the effect is converging from lower levels of environmental degradation towards higher levels in Bulgaria, Croatia, Czech, Estonia, Latvia, Lithuania, Poland, Romania, Slovenia, and Slovakia. Countries like Austria, Belgium, Denmark, Finland, France, Italy, and Luxembourg have stable effect, which means it does not vary over time.

The effect of research and development expenses is higher in Romania, Bulgaria, Slovakia Latvia, and Luxembourg, whereas the lowest effect is measured in Germany, Finland, France, Austria, and Belgium. Temporal graph verifies the idea that research and development converge the environmental degradation to lower level. Austria, Belgium, Czech, Denmark, Finland, France, Italy, Poland, Spain, and Sweden all show a downward behavior in environmental degradation on introduction of research and development.

## Discussion and concluding remarks

Upon exploring the relationship between stock market growth, economic strength, R&D expenditures, and environmental degradation, we find that the stock markets growth can cause an upward swing in the environmental degradation in the longer run, and this behavior is opposite in the short run. The change in behavior can be explained as the sustainable development goals (SDGs) were established in 2015 and the pre-SDG period is more dominant in this research. This explains ceteris paribus after the establishment of the SDGs the firms’ focus and demand changed to renewable energy resources and firms’ themselves focused on becoming environmentally friendly (contributing less to the environmental degradation). These results are in line with the work of Mhadhbi et al. (2021) and Shahbaz et al. (2020a) as their work also finds that the financial development over a longer period of time contributes towards the environmental degradation. From the context of SDGs, stock markets’ growth should reduce the environmental degradation. Economic strength in terms of long run contributes towards the environmental degradation; however, in the short run, only the diminishing aspect can increase the environmental degradation. This might be explained as the economic strength lowers, countries are found with scarce resources to support the SDGs and contribute towards the environmental degradation. Research and development expenditures’ symmetric evidences is in line with work of Alam et al. (2019), Ganda (2019), Jin et al. (2017), and Shahbaz et al. (2020a) as they find that the R&D expenditure lowers the environmental degradation. A profound explanation can be that when a country invests in newer technology’s research the scale is initially small; however, with time, the production scale on the new technology increases (Awaworyi Churchill et al. 2019). Even if the newer technology is environmentally friendly, the production of it might not be, as it is evident from this research’s results that firms in longer run prompt the environmental degradation. This shows that the environmental degradation shifts from one form to another.

The policy implications are two-fold in this study. Firstly, the shift of environmental degradation from one form to another form is critical for the policy making. The elimination of environmental degradation should be in full form rather than the illusion of stopping the climate change. Secondly, the policy makers should focus on converging the stock market growth short-run effect to the longer run. The financial markets are an essential part of an economy, it would be optimum if the financial markets can lower the environmental degradation. Recent and upcoming events, such as COVID-19 and the Ukraine-Russia war, as well as their implications on economic growth and the likely policy reaction, pose a number of challenges for the economy and the environment. These challenges are likely to be exacerbated by the implications of these events. In terms of the policies that should be implemented, what this indicates is that countries should strive to make the economy to achieve SDGs by 2030. EU should promote and support environmentally friendly initiatives. In such situation, the market should finance environmentally (SDGs) friendly projects. As a consequence, more expenditures for R&D may support environment. Finally, the increase of capital market and R&D expenditures could have positive impact on environment.

## Data Availability

Available on request.

## Appendix

**Table 7:** Stock market indices information

Country	Symbol	Index
Austria	ATX	Austrian Traded Index
Belgium	BFX	Brussels 20
Bulgaria	BSE SOFIX^1^	SOFIX Index
Croatia	CROBEX^2^	Zagreb Stock Index
Cyprus	CYMAIN	Cyprus Main Market
Czech Republic	PX^3^	Prague Stock Market Index
Denmark	OMXCGI^4^	OMX Copenhagen All
Estonia	OMXTGI	OMX Tallinn GI
Finland	OMXHPI	OMX Helsinki
France	CAC	CAC ALL
Germany	DAX	DAX ALL
Greece	ATG	Athens General Composite Index
Hungry	BUX^5^	Budapest SE
Ireland	ISEQ	ISEQ Overall
Italy	MIB	FTSE MIB
Latvia	OMXRGI	OMX Riga GI
Lithuania	OMXVGI	OMX Vilnius GI
Luxembourg	LUXX	LuXX Index
Malta	MSE	Malta Stock Exchange Index
Netherlands	AEX	Amsterdam Stock Market All Index
Poland	WIG^6^	Poland Stock Exchange Index
Portugal	PSI20	PSI 20
Romania	BET^7^	Bucharest Stock Exchange Trading Index
Slovakia	SAX	Slovakia SAX Index
Slovenia	SBITOP	Blue Chips SBITOP
Spain	IBEX	IBEX 35
Sweden	OMX Stockholm^8^	OMX Stockholm 30

Currency of index is EUR except those mentioned below:

^1^BGN, ^2^HRK, ^3^CZK, ^4^DKK, ^5^HUF, ^6^PLN, ^7^BET, and ^8^SEK

*Own elaboration*

## Footnotes

1 Some variables of this study are integrated at *I(1)* and ARDL would produce robust results.
